# Integrative genomic profiling identifies MLPH as a candidate gene in prostate cancer

**DOI:** 10.3389/fmed.2026.1878505

**Published:** 2026-07-10

**Authors:** Runyi Wang, Jiayu Wang, Zhiyi Zhao, Xiaopeng Hu

**Affiliations:** 1 Department of Urology, Beijing Chao-Yang Hospital, Capital Medical University, Beijing, China; 2 Institute of Urology, Capital Medical University, Beijing, China

**Keywords:** colocalization, genome-wide association studies (GWAS), MLPH, prostate cancer, transcriptome-wide association studies (TWAS)

## Abstract

**Background:**

Prostate cancer (PCa) is a highly heterogeneous malignancy with complex genetic underpinnings. This study integrates multi-omics data to prioritize candidate susceptibility genes and evaluate their functional and clinical significance in PCa pathogenesis.

**Materials and methods:**

We integrated PCa GWAS summary statistics with GTEx v8 expression quantitative trait locus reference panels to perform cross-tissue and single-tissue transcriptome-wide association studies. Candidate signals were refined using conditional analysis, MAGMA and fastBAT gene-level tests, Summary data-based Mendelian randomization, and Bayesian colocalization. Tumor-context cis-eQTL evidence from TCGA-PRAD was incorporated to prioritize regulatory signals retained in prostate cancer tissues. Prioritized candidates were further assessed using transcriptomic datasets, Human Protein Atlas immunohistochemistry, single-cell RNA-seq analysis, histological grading, preoperative PSA, and established genomic risk signatures. Gene network and pathway enrichment analyses were performed to explore potential biological context.

**Results:**

The integrative genetic analyses identified 23 consensus candidate genes supported by multiple association frameworks. SMR and Bayesian colocalization further narrowed the candidate list, and tumor-context cis-eQTL analysis in TCGA-PRAD retained *MLPH* as the final prioritized candidate. The lead variant rs7582964 was significantly associated with *MLPH* expression in PRAD tumor tissues. MLPH was upregulated in PCa tissues compared with normal prostate tissues in TCGA-PRAD and showed concordant expression patterns in an independent GEO cohort and Human Protein Atlas immunohistochemistry data. Single-cell transcriptomic analysis localized MLPH expression mainly to epithelial cells, with the strongest signal observed in tumor epithelial cells. Clinically, *MLPH* expression was associated with histological differentiation, with reduced expression in the most poorly differentiated tumors. Lower *MLPH* expression also correlated with higher preoperative PSA and higher Decipher-like genomic risk scores. Functional analyses linked MLPH to vesicle-mediated transport, exocytosis, and hormone-related signaling pathways.

**Discussion and conclusion:**

This integrative genomic analysis prioritizes *MLPH* as a candidate susceptibility gene for PCa and links its regulatory signal to tumor-context expression, protein-level evidence, cellular localization, and clinically relevant molecular features. These findings support a potential role for MLPH in PCa biology, particularly in relation to vesicle trafficking and tumor differentiation.

## 1 Introduction

Prostate cancer (PCa) remains a major global health burden and one of the most frequently diagnosed malignancies among men worldwide. According to GLOBOCAN 2022 estimates, PCa accounted for approximately 1.47 million new cases worldwide and ranked as the second most commonly diagnosed cancer in men (1). In the United States, 333,830 new PCa cases and 36,320 deaths are projected to occur in 2026 (2). Early-stage PCa is often clinically silent, whereas advanced disease may present with urinary symptoms, bone pain, or other manifestations related to local progression or metastatic spread (3). Despite improvements in screening, risk stratification, and systemic therapy, a substantial subset of patients still develop aggressive or metastatic disease. These observations underscore the need to clarify the genetic and regulatory mechanisms underlying PCa susceptibility and progression.

Genetic predisposition plays a significant role in the pathogenesis of PCa, which is considered one of the most heritable cancers (4–6). To date, genome-wide association studies (GWAS) have identified over 170 risk loci associated with PCa susceptibility (7, 8). However, the biological mechanisms by which these loci influence tumor development remain largely unresolved. A substantial number of the identified risk variants reside in noncoding regions, complicating functional interpretation (9). Furthermore, complex patterns of linkage disequilibrium (LD) often hinder the precise localization of candidate variants (10).

Transcriptome-wide association studies (TWAS) have emerged as a powerful approach to prioritize candidate genes by integrating expression quantitative trait loci (eQTL) information with GWAS summary statistics (11). Compared with traditional GWAS, TWAS improves gene-level resolution and offers insight into the regulatory architecture of complex traits (10). Particularly, cross-tissue TWAS frameworks such as the unified test for molecular signatures (UTMOST) (12) enhance detection power by leveraging shared eQTL effects across tissues while preserving tissue-specific signals via a group-lasso penalty model (13). This cross-tissue approach has demonstrated utility in multiple complex diseases, including osteoarthritis, erectile dysfunction, Alzheimer’s disease and age-related macular degeneration (14–17).

In this study, we integrated GWAS summary statistics for PCa from the GWAS Catalog with eQTL data from the Genotype-Tissue Expression (GTEx) project (version 8) (18) to conduct TWAS. To systematically identify PCa–associated genes, we applied both cross-tissue and single-tissue TWAS, followed by conditional analysis and complementary gene-level association testing. Putative causal associations between gene expression and disease risk was assessed using Summary data-based Mendelian randomization (SMR) and Bayesian colocalization (19, 20). We then incorporated tumor-context cis-eQTL evidence from TCGA-PRAD through PancanQTL to prioritize regulatory signals retained in prostate cancer tissues. The final candidate was subsequently assessed using independent transcriptomic, protein-level, and clinical annotation resources, followed by gene network and pathway enrichment analyses to explore its biological context in PCa.

## 2 Methods

### 2.1 PCa GWAS data sources

GWAS data for PCa were obtained from a large multi-ancestry meta-analysis study (21). To reduce potential confounding from population stratification, we only selected the European-ancestry subset for downstream analyses. This subset, indexed as GCST90274714 in the GWAS Catalog, comprises 122,188 prostate cancer cases and 604,640 controls.

### 2.2 TWAS analyses in cross-tissue

**Figure 1:**
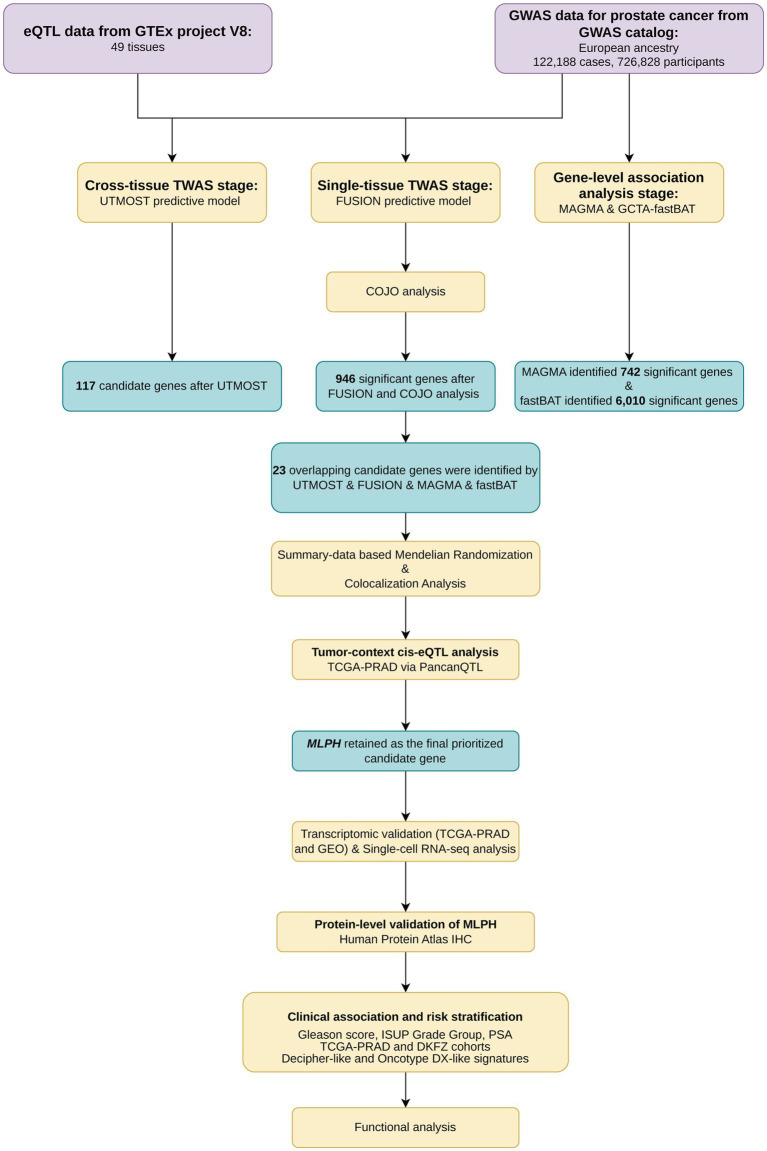
The overall study framework. GWAS, genome-wide association studies; TWAS, transcriptome-wide association studies; GTEx, Genotype-Tissue Expression; UTMOST, unified test for molecular signatures; FUSION, Functional Summary-based Imputation; MAGMA, Multi-marker Analysis of Genomic Annotation; fastBAT, fast set-Based Association Test; GCTA, Genome-wide Complex Trait Analysis; COJO, conditional and joint analysis; TCGA, The Cancer Genome Atlas; HPA, Human Protein Atlas; ISUP, International Society of Urological Pathology.

Transcriptome-wide association studies framework employed in this study is illustrated in Figure 1. During the discovery phase, we utilized the UTMOST framework, which leverages multi-tissue eQTL data to assess gene–trait associations at a systemic level (12). This approach incorporates eQTL effect estimates derived from 49 tissues in the GTEx V8 dataset, enabling the construction of cross-tissue predictive models for individual genes. By integrating across tissues, UTMOST improves both the power to detect gene–phenotype associations and the accuracy of expression imputation, particularly in tissues enriched for trait heritability. To aggregate tissue-specific associations, we implemented the generalized Berk-Jones (GBJ) test (22), which accounts for correlation structures among tissues using covariance estimates from single-tissue summary statistics. Statistical significance was defined using two thresholds: genes reaching an FDR of < 1 × 10^−5^ were considered genome-wide significant after correction for multiple testing, while those with a *p*-value < 0.01 were considered to reach nominal significance (16).

### 2.3 TWAS analyses in single-tissue and conditional analysis

We conducted a TWAS for PCa by integrating GWAS statistics with eQTL data from 49 tissues available in the GTEx V8 reference panel, utilizing the functional summary-based imputation (FUSION) pipeline (23). LD patterns between SNPs and expression prediction models were inferred using European samples from the 1,000 Genomes Project (24). FUSION assessed several modeling approaches for imputing gene expression and selected the optimal model based on cross-validation performance (25). The resulting gene expression weights were then integrated with PCa GWAS Z-scores to estimate gene–trait associations. Genes were retained for further investigation if they met both of the following thresholds: (1) an FDR < 0.05 in the single-tissue analysis, and (2) Bonferroni-corrected *p* < 0.05 in at least one individual tissue analysis. To evaluate whether these associations identified in single-tissue TWAS are conditionally independent, we performed conditional and joint analysis (COJO) using FUSION software package (26). This analysis accounts for LD among variants, enabling a more refined interpretation of the genetic architecture underlying trait variation. Genes that retained statistical significance after conditioning were classified as jointly significant, whereas those that lost significance were considered marginally significant (27). In line with the FUSION pipeline, we applied a correlation filter of TOP. SNP. COR^2^ ≤ 0.6 to exclude genes whose lead SNPs exhibited high LD with conditioned variants, thereby reducing redundancy and enhancing signal independence. Only genes that were jointly significant and satisfied this LD threshold were retained for downstream analysis.

### 2.4 MAGMA-based gene association analysis

Gene-level association analysis was performed using Multi-marker Analysis of Genomic Annotation (MAGMA) (v1.08), a widely used tool for evaluating gene-based associations from GWAS summary data (28). MAGMA identifies trait-associated genes by aggregating the effects of multiple SNPs assigned to each gene based on their genomic location. In this study, we used the default parameters to derive gene-level association statistics from SNP-level data (29, 30). Genes reaching the Bonferroni-corrected significance threshold (*p* < 0.05) were considered statistically significant and were regarded as potential contributors to the trait (28, 31).

### 2.5 Gene-based association analysis using GCTA-fastBAT

Gene-based association analysis for prostate cancer was conducted using the fastBAT module implemented in the GCTA software (32, 33). This method aggregates SNP-level z-statistics within a gene region into a composite test statistic, while accounting for LD among variants (34). By modeling the correlation structure of nearby SNPs, fastBAT efficiently estimates the joint significance of multiple variants mapped to a gene.

### 2.6 SMR analyses and Bayesian colocalization

We applied SMR to explore potential pleiotropic associations between gene expression and prostate cancer risk, using integrated summary statistics from PCa GWAS and eQTL datasets. This method enables the prioritization of genes that may be putatively associated with disease risk, thereby offering insights into the functional basis of GWAS signals (35). SMR associations were considered significant if they met an FDR-adjusted *p* < 0.05, while evidence of heterogeneity was assessed using the heterogeneity in dependent instruments (HEIDI) test, with *p* > 0.01 indicating no significant heterogeneity (19). To visualize the effect sizes and confidence intervals of candidate genes identified by SMR, we used the “forestplot” R package (v3.1.6) to generate a forest plot. To further evaluate the likelihood of a shared genetic variant underlying both eQTL and GWAS signals, we performed Bayesian colocalization analysis using the coloc package in R (v5.2.3) (20). This method computes posterior probabilities for five mutually exclusive hypotheses: H0, no association with either trait; H1, association with the first trait only; H2, association with the second trait only; H3, association with both traits but driven by different genetic variants; and H4, association with both traits driven by the same genetic variant. Evidence supporting colocalization was defined as a posterior probability for hypothesis 4 (PP.H4) > 0.70 (36).

### 2.7 Tumor-context *cis*-eQTL prioritization using PancanQTL

To evaluate whether genetically prioritized candidate signals were retained in the prostate cancer tumor context, we queried cis-eQTL associations in TCGA-PRAD using PancanQTL (37). Candidate genes emerging from SMR and Bayesian colocalization were assessed together with their lead regulatory variants. Genes were retained for downstream transcriptomic, protein-level, and clinical characterization when the corresponding lead variant showed a significant cis-eQTL association with gene expression in PRAD tumor tissues. This step was used as a conservative tumor-context prioritization filter rather than as definitive exclusion of genes without detectable tumor cis-eQTL evidence.

### 2.8 External validation with TCGA-PRAD data and GEO cohorts

RNA sequencing and clinical data for prostate adenocarcinoma (PRAD) were obtained from the UCSC Xena platform (38), comprising 554 prostate tissue samples from The Cancer Genome Atlas (TCGA). Of these, 483 were tumor samples. To assess the robustness of our findings in an independent patient population, we additionally retrieved the GSE221107 dataset from the Gene Expression Omnibus (GEO), which includes 20 prostate cancer tissues and 20 matched paracarcinoma normal tissues. Differential gene expression analysis for both cohorts was conducted using the DESeq2 package in R (v1.46.0) (39). *p*-values were corrected for multiple testing using the Benjamini–Hochberg method. Genes with an adjusted *p* < 0.05 and an absolute log₂ fold change (|log₂FC|) > 0.58 were considered differentially expressed (40). Visualization of results was carried out using “ggplot2” R package (v3.5.2).

### 2.9 Protein-level validation in clinical prostate tissues

To validate protein-level expression of candidate genes, immunohistochemistry (IHC) data were obtained from the Human Protein Atlas (HPA; Antibody ID: HPA014685) (41, 42). Representative IHC images of PCa and normal prostate tissues were reviewed. For semi-quantitative assessment, three non-overlapping regions of interest were randomly selected from each image, and the immunopositive area was measured using ImageJ through the Fiji platform (43). The mean value of the three regions was used to represent protein expression for each specimen. Because of the limited number of available IHC images, differences between PCa and normal prostate tissues were assessed using a two-sided Mann–Whitney *U* test. A *p*-value < 0.05 was considered statistically significant. Statistical analysis and visualization were performed using GraphPad Prism 10.

### 2.10 Single-cell RNA-seq analysis

To assess the cellular distribution of the final prioritized candidate gene, we analyzed the publicly available prostate cancer single-cell RNA-seq dataset GSE181294 (44). Author-provided cell-type annotations were retained as the primary labels. A condition- and cell-type-stratified subset was processed using Seurat. Raw counts were log-normalized, highly variable genes were identified, and scaled data were used for principal-component analysis. Sample-level batch effects were corrected using Harmony, and UMAP embedding was generated from the corrected dimensions. Fine cell-type annotations were collapsed into major cell classes for overview visualization and marker validation, while the original fine annotations were retained for tissue condition–specific localization. Candidate-gene expression was summarized using log-normalized expression and the proportion of cells with detectable expression. Sample-level epithelial summaries were calculated for sample–cell-type strata containing at least 20 retained cells.

### 2.11 Clinical stratification and prognostic signature analysis

To investigate the clinical relevance of the final prioritized candidate gene in the context of tumor differentiation and risk stratification, we utilized clinical annotations from the TCGA-PRAD cohort and the independent DKFZ validation cohort (45). Patients were stratified based on pathological Gleason scores and reclassified into International Society of Urological Pathology (ISUP) Grade Groups (1–5) according to the 2014 ISUP consensus standards (46). Differences in candidate-gene expression across ISUP Grade Groups were assessed using the Kruskal-Wallis test, followed by pre-specified pairwise Wilcoxon rank-sum tests. The association between candidate-gene expression and preoperative PSA levels in the DKFZ cohort was evaluated using Spearman’s rank correlation analysis. Decipher-like and Oncotype DX-like signature scores were calculated as the mean normalized expression z-scores of available constituent genes in each panel (47, 48). Correlations between candidate-gene expression and these signature scores were assessed using Spearman correlation analysis in both the TCGA-PRAD and DKFZ cohorts. Survival analyses were additionally performed in cohorts with available follow-up data. Kaplan–Meier curves with log-rank tests compared high and low expression groups for the final prioritized candidate gene using cohort-specific median cutoffs, and Cox proportional hazards models evaluated z-scored continuous candidate-gene expression with adjustment for available clinicopathological covariates.

### 2.12 Gene network, functional enrichment, and exploratory perturbagen analysis

We employed the GeneMANIA platform, a network-based tool that integrates diverse genomic and proteomic datasets, including co-expression, genetic interactions, and pathway annotations (49). To further investigate the functional context of genes prioritized by GeneMANIA, we conducted gene set enrichment analysis using the clusterProfiler R package (v4.14.6) (50). Enrichment was assessed across multiple curated annotation resources, including Gene Ontology (GO), Kyoto Encyclopedia of Genes and Genomes (KEGG) and Reactome databases, to identify biological processes and pathways significantly enriched among the input gene set (51–53). As an exploratory translational annotation of the 23 consensus genes, the TCGA-PRAD-derived directional candidate-gene signature was queried in L1000CDS2 to identify perturbagens predicted to reverse the expression pattern, and DGIdb was queried separately to annotate known drug-gene interactions.

## 3 Results

### 3.1 TWAS identifies cross-tissue PCa-associated genes

In the cross-tissue TWAS analysis performed using the UTMOST framework, 240 genes reached the nominal significance threshold (*p* < 0.01), among which 117 genes surpassed the genome-wide significance level (*p* < 1 × 10^−5^) (Supplementary Table S1). These results provide initial evidence for transcriptome-wide associations with PCa across multiple tissues.

### 3.2 Single-tissue TWAS and COJO analysis identify independent loci

Single-tissue TWAS identified 73 genes that overlapped with cross-tissue TWAS results in at least one GTEx tissue using a Bonferroni-corrected threshold (*p* < 0.05) (Supplementary Table S2). To evaluate the independence of these associations, we conducted COJO on the significant genes identified in single-tissue TWAS, identifying 946 that remained jointly significant. Among these, 35 genes overlapped with the 117 genome-wide significant genes from the UTMOST cross-tissue TWAS (Table 1), suggesting these represent robust and tissue-consistent associations. These genes were primarily located on chromosomes 2, 4, and 22, indicating the presence of gene-dense regions with potential biological relevance to PCa.

**Table 1:** Candidate genes identified by cross-tissue and single-tissue TWAS after conditional analysis.

Gene_id	Gene	CHR	FDR	Start	End
ENSG00000249264	EEF1A1P9	4	3.45E-10	106405855	106407237
ENSG00000162882	HAAO	2	6.43E-10	42994229	43019733
ENSG00000223374	AC005104.3	2	8.16E-10	242290755	242292519
ENSG00000003400	CASP10	2	1.92E-09	202047604	202094129
ENSG00000100290	BIK	22	1.92E-09	43506754	43525718
ENSG00000100294	MCAT	22	1.92E-09	43528212	43539400
ENSG00000101751	POLI	18	1.92E-09	51795774	51847636
ENSG00000115486	GGCX	2	1.92E-09	85771846	85788670
ENSG00000115504	EHBP1	2	1.92E-09	62900986	63273622
ENSG00000115648	MLPH	2	1.92E-09	238394071	238463961
ENSG00000115761	NOL10	2	1.92E-09	10710892	10830101
ENSG00000138777	PPA2	4	1.92E-09	106290234	106395238
ENSG00000152256	PDK1	2	1.92E-09	173420101	173489823
ENSG00000152518	ZFP36L2	2	1.92E-09	43449541	43453748
ENSG00000163106	HPGDS	4	1.92E-09	95219686	95264027
ENSG00000168385	SEPT2	2	1.92E-09	242254515	242293442
ENSG00000168769	TET2	4	1.92E-09	106067032	106200973
ENSG00000168883	USP39	2	1.92E-09	85829979	85876403
ENSG00000184058	TBX1	22	1.92E-09	19744226	19771116
ENSG00000236699	ARHGEF38	4	1.92E-09	106473777	106629250
ENSG00000169435	RASSF6	4	2.42E-08	74437267	74486348
ENSG00000091409	ITGA6	2	7.52E-08	173292082	173371181
ENSG00000145391	SETD7	4	0.000000101	140417095	140527853
ENSG00000064012	CASP8	2	0.000000118	202098166	202152434
ENSG00000198612	COPS8	2	0.000000378	237993955	238009109
ENSG00000176635	HORMAD2	22	0.000000429	30476163	30573064
ENSG00000132466	ANKRD17	4	0.000000462	73939093	74124515
ENSG00000231609	AC009501.4	2	0.000000707	63271057	63275775
ENSG00000003402	CFLAR	2	0.00000117	201980827	202041410
ENSG00000115677	HDLBP	2	0.00000145	242166679	242256476
ENSG00000100300	TSPO	22	0.00000169	43547520	43559248
ENSG00000196290	NIF3L1	2	0.0000021	201754050	201768655
ENSG00000248641	HMGA1P2	4	0.00000337	73964539	73964862
ENSG00000057935	MTA3	2	0.00000608	42721709	42984087
ENSG00000100325	ASCC2	22	0.00000673	30184597	30234271

FDR, false discovery rate; CHR, chromosome.

### 3.3 Gene-level association and burden tests support convergent loci

**Figure 2:**
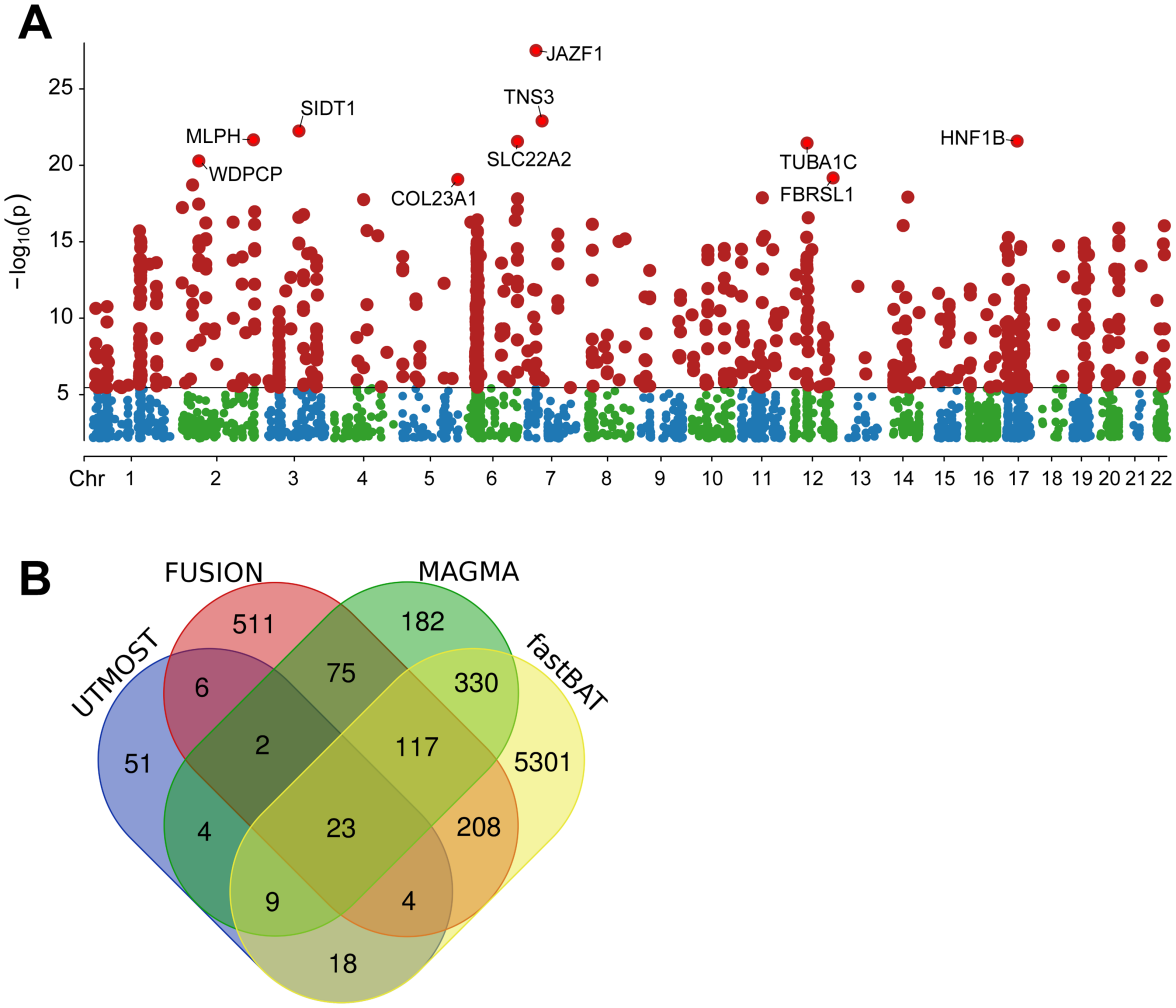
Gene-level association and integrative prioritization of prostate cancer susceptibility genes. **(A)** Manhattan plot of gene-based association results from MAGMA. Each point represents a gene, plotted according to its chromosomal position on the *X*-axis and −log₁₀(P) value on the *Y*-axis, derived from the *z*-score test. The top 10 most significant genes are annotated directly on the plot. A total of 742 genes reached statistical significance following Bonferroni correction (*p* < 0.05). **(B)** Venn diagram summarizing the intersection of candidate genes identified by four independent analytical frameworks: MAGMA, fastBAT, UTMOST, and FUSION TWAS. Twenty-three genes were consistently prioritized across all methods. MAGMA, Multi-marker Analysis of Genomic Annotation; fastBAT, fast set-Based Association Test; UTMOST, unified test for molecular signatures; FUSION, Functional Summary-based Imputation; TWAS, Transcriptome-wide association studies.

MAGMA gene-level analysis identified 742 genes significantly associated with PCa after Bonferroni correction (*p* < 0.05; Supplementary Table S4). The top ten genes with the most significant associations are annotated in the Manhattan plot (Figure 2A). MAGMA-based pathway enrichment analysis revealed significant enrichment of transmembrane transport pathways (Supplementary Figure S1A). Tissue-specific enrichment further pointed to the prostate as the most relevant tissue, with additional signals observed in minor salivary gland, vagina, and esophagus (Supplementary Figure S1B), supporting an epithelial origin for prostate cancer susceptibility. Additionally, fastBAT gene-based burden testing identified 6,010 genes with significant associations (*p* < 0.05). Among these, 46 genes overlapped with the results from both cross-tissue and single-tissue TWAS analyses, highlighting their potential relevance to PCa and providing complementary evidence to the TWAS and MAGMA findings (Supplementary Table S5).

### 3.4 Integrated analysis prioritizes 23 high-confidence candidate genes

To refine our candidate gene list, we intersected the jointly significant genes from single-tissue TWAS (via COJO), cross-tissue TWAS results, MAGMA gene-level associations, and fastBAT burden signals. This integrative approach yielded a consensus set of 23 genes with multilayered statistical support for PCa involvement: *MCAT*, *HORMAD2*, *SEPT2*, *USP39*, *ZFP36L2*, *MTA3*, *HAAO*, *ANKRD17*, *GGCX*, *CFLAR*, *CASP10*, *TBX1*, *TSPO*, *TET2*, *RASSF6*, *MLPH*, *BIK*, *HDLBP*, *EHBP1*, *PPA2*, *NOL10*, *CASP8*, and *PDK1* (Figure 2B). These genes represent strong candidates for further investigation at the functional and clinical levels. We therefore performed an exploratory L1000CDS2 reverse-signature query using the TCGA-PRAD directional expression pattern of these 23 genes. The top-ranked perturbagens included piperlongumine, JAK3 Inhibitor VI, cymarin, doxorubicin hydrochloride, MLN4924, trichostatin A, and GR-109; DGIdb provided drug-gene interaction annotations for 10 consensus genes but did not identify a direct MLPH-drug interaction (Table S9).

### 3.5 SMR and colocalization analyses refine the prioritization of candidate genes

**Figure 3:**
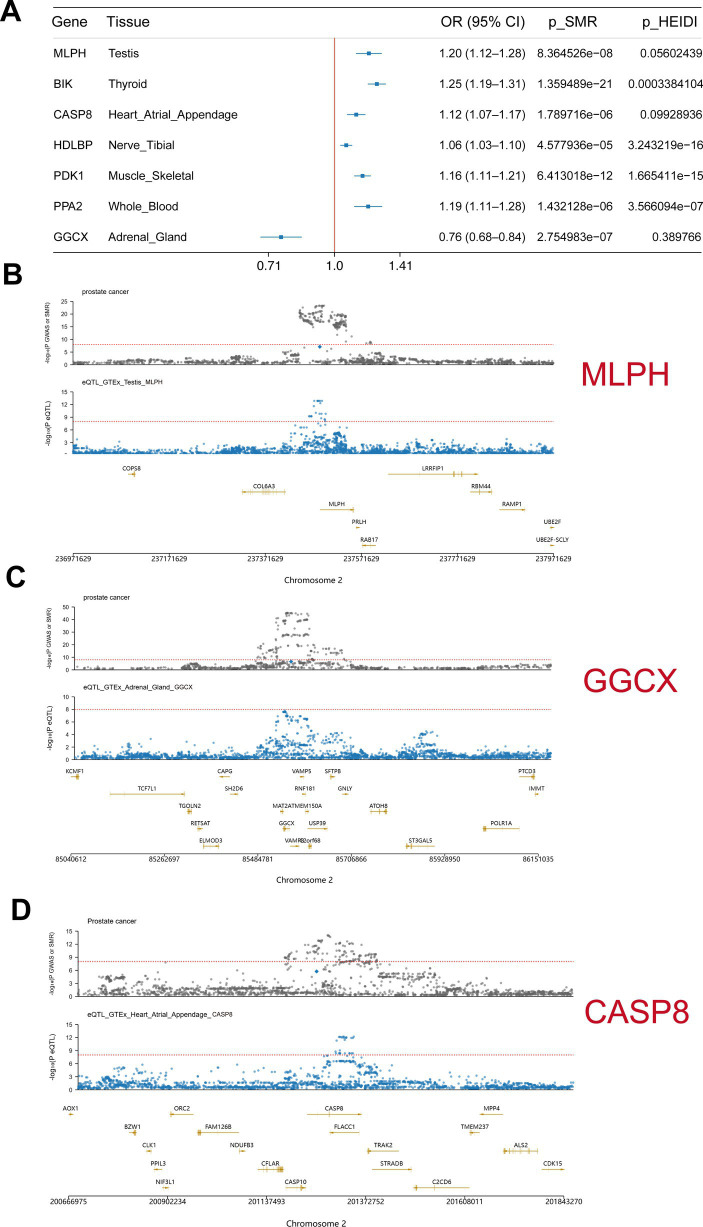
SMR analysis prioritizes putative candidate genes for prostate cancer. **(A)** Forest plot summarizing SMR results for seven genes across multiple tissues. ORs with 95% CIs are shown, along with *P*_SMR and *p*_HEIDI values for each tissue. *MLPH*, *GGCX* and *CASP8* surpassed the SMR significance threshold (FDR-adjusted *P*_SMR < 0.05) and showed no evidence of heterogeneity (*p*_HEIDI > 0.01), supporting potential shared genetic variants. **(B–D)** Regional plots illustrating SMR results for *MLPH* **(B)**, *GGCX* **(C)**, and *CASP8* **(D)**. Each dot represents a SNP. Top SNPs used in the SMR analysis are highlighted. SMR, summary-data-based Mendelian randomization; HEIDI, heterogeneity in dependent instruments; OR, odds ratio; CI, confidence interval; SNP, single nucleotide polymorphism.

To further prioritize putative candidate genes, we conducted SMR and colocalization analyses. For each candidate gene, we used eQTL data from the GTEx V8 tissues in which the gene was significant in single-tissue TWAS combined with COJO results, together with the PCa GWAS summary statistics. SMR analysis initially identified seven genes with *P*_SMR < 0.05, indicating significant associations between genetically predicted expression and PCa risk (Figures 3A–D and Supplementary Table S6). Of these, only three genes—*MLPH*, *CASP8*, and *GGCX*—also passed the HEIDI heterogeneity test (*p*_HEIDI > 0.01), suggesting that their associations are unlikely to arise from LD with neighboring variants and may represent shared genetic signals between gene expression and disease susceptibility.

**Figure 4:**
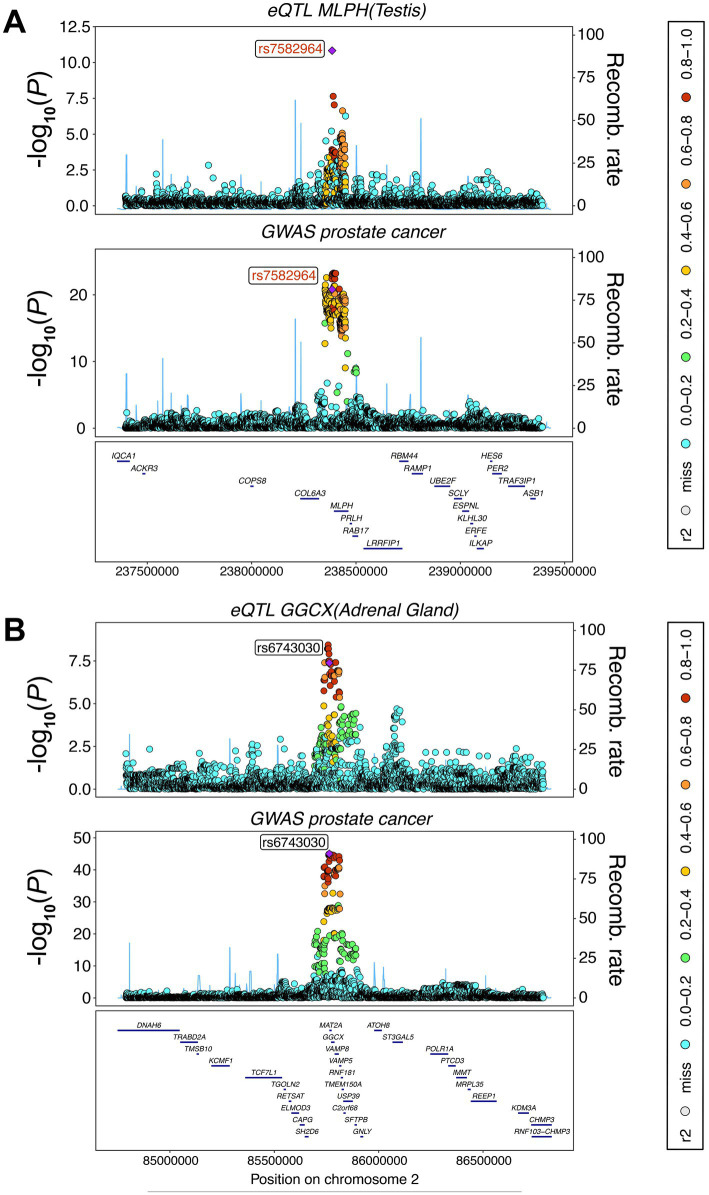
Colocalization results. **(A)** For *MLPH* in testis tissue, the lead SNP rs7582964 shows strong association with both gene expression (top) and disease risk (bottom), with a posterior probability of colocalization (PP.H4 = 0.761), indicating a likely shared genetic variant. **(B)** For *GGCX* in adrenal gland tissue, the lead SNP rs6743030 also shows colocalized association peaks (PP.H4 = 0.968).

To validate these findings, we performed colocalization analysis. Among the 3 genes tested, two—*MLPH* and *GGCX*—showed suggestive or strong support for a shared variant (PP.H4 > 0.70; Figure 4, Supplementary Figure S2, and Supplementary Table S7). For *MLPH*, the PP.H4 reached 0.761 in the testis, indicating strong colocalization of GWAS and eQTL signals in testis tissue. Notably, rs7582964 was identified as the top SNP in the SMR and emerged as the key colocalized site for *MLPH* in testis tissue, reinforcing the likelihood that this variant serves as a shared candidate driver at this locus. For *GGCX*, strong colocalization signals were observed at rs6743030 in adrenal gland tissue.

### 3.6 Tumor-context *cis*-eQTL analysis supports MLPH as the final prioritized candidate

**Figure 5:**
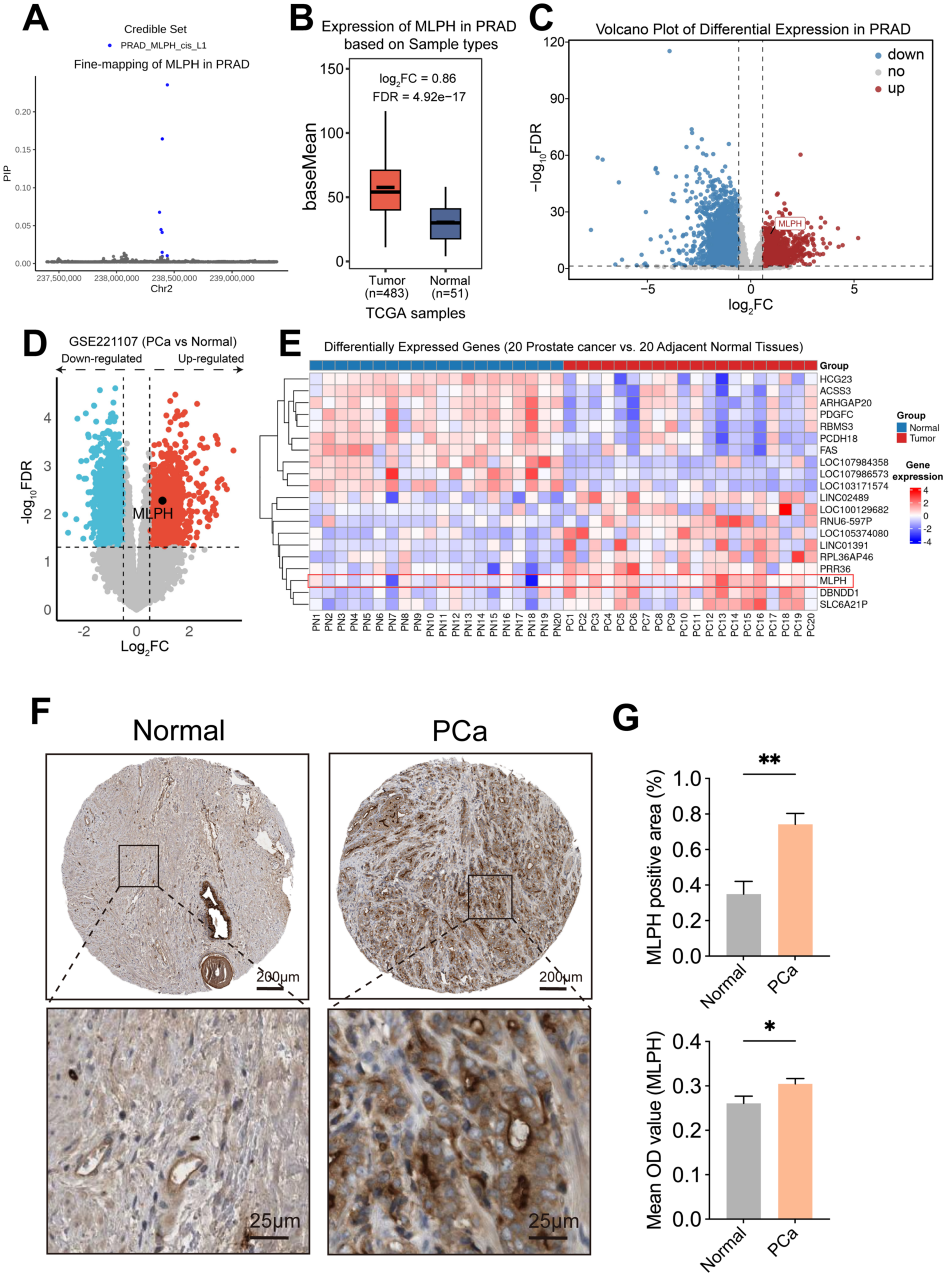
Multi-cohort validation of MLPH upregulation at genetic, transcriptomic, and protein levels. **(A)** *Cis*-eQTL association between the lead variant rs7582964 and *MLPH* expression in the TCGA-PRAD cohort (*p* = 0.045). **(B,C)** Transcriptional validation in the TCGA-PRAD cohort. **(B)** Boxplot showing significant upregulation of *MLPH* in tumor tissues (*n* = 483) compared with normal controls (*n* = 51) (log_2_FC = 0.86, FDR = 4.92 × 10^−17^). **(C)** Volcano plot highlighting *MLPH* among significantly upregulated genes. **(D,E)** Independent validation in the GSE221107 cohort. **(D)** Volcano plot and **(E)** heatmap confirming robust and consistent *MLPH* mRNA upregulation in tumor samples compared to matched paracarcinoma tissues. **(F,G)** Protein-level validation using Human Protein Atlas (HPA) data. **(F)** Representative immunohistochemical (IHC) staining and **(G)** quantitative assessment of positive area (%) and mean optical density (OD), demonstrating significantly increased MLPH protein expression in prostate cancer tissues. TCGA, The Cancer Genome Atlas; PRAD, prostate adenocarcinoma; GEO, Gene Expression Omnibus; HPA, Human Protein Atlas; PCa, prostate cancer. Images in panel **F** were adapted from the Human Protein Atlas (MLPH, antibody HPA014685; image credit: Human Protein Atlas; licensed under Creative Commons Attribution 4.0 International License). In panel **G**, *p < 0.05; **p < 0.01 (41).

Following SMR and Bayesian colocalization analyses, *MLPH* and *GGCX* remained as the two candidate genes with convergent genetic evidence. To determine whether these regulatory signals were retained in the prostate cancer tumor context, we next queried cis-eQTL associations in TCGA-PRAD using PancanQTL. The lead variant for *MLPH*, rs7582964, showed a significant association with *MLPH* expression in PRAD tumor tissues (*p* = 0.045; Figure 5A), indicating detectable tumor-context regulatory evidence for this locus. In contrast, no significant cis-eQTL association was observed for *GGCX* or its lead SNP rs6743030 in PRAD. Therefore, *MLPH* was retained as the final prioritized candidate for downstream transcriptomic, protein-level, and clinical analyses.

### 3.7 Multi-cohort validation of MLPH expression at the transcriptomic and protein levels

After tumor-context cis-eQTL analysis retained MLPH as the final prioritized candidate, we evaluated its expression pattern across independent transcriptomic and protein-level resources. In TCGA-PRAD, MLPH was significantly upregulated in prostate cancer tissues compared with normal prostate tissues, with a log₂FC of 0.86 and an adjusted *p* value of 4.92 × 10^−17^ (Figures 5B,C). This tumor-associated upregulation was further supported in the independent GSE221107 cohort, in which MLPH expression was higher in prostate cancer tissues than in matched paracarcinoma tissues (Figures 5D,E). At the protein level, immunohistochemical data from the Human Protein Atlas showed stronger MLPH staining in prostate cancer tissues than in normal prostate tissues (Figures 5F,G), consistent with the transcriptomic findings.

### 3.8 Single-cell localization of MLPH expression in prostate epithelial compartments

**Figure 6:**
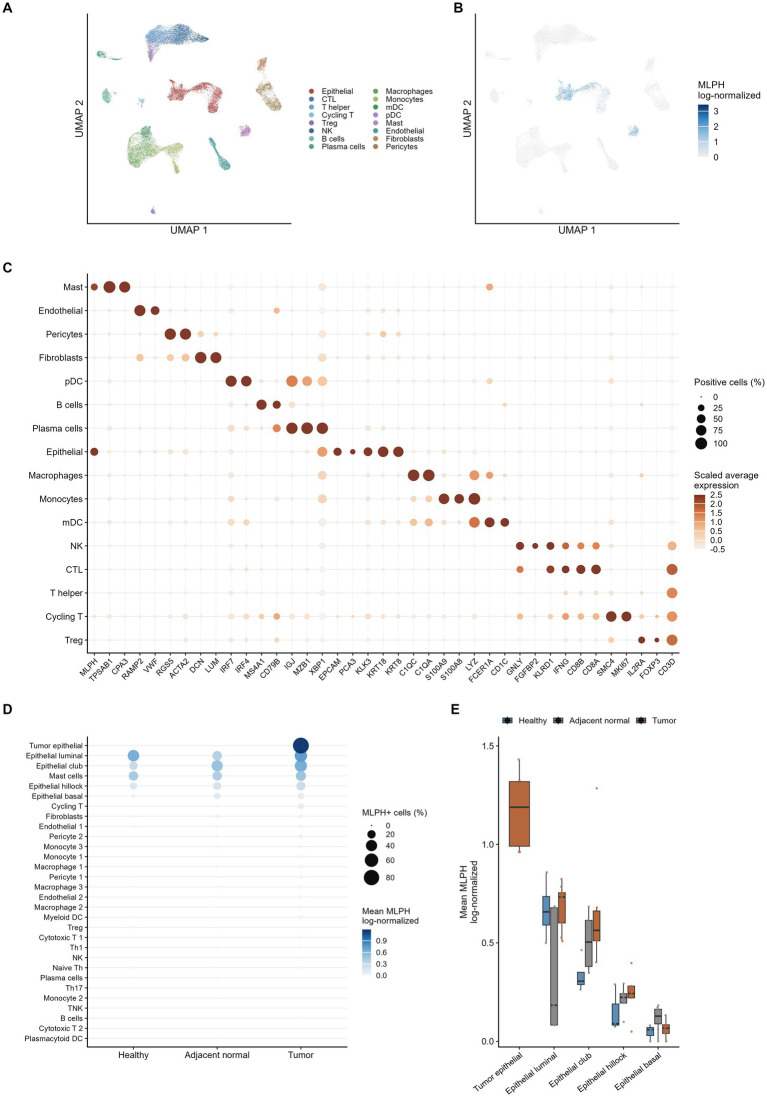
Single-cell localization of MLPH expression in prostate cancer. **(A)** UMAP representation of prostate single-cell RNA-seq data from GSE181294, colored by author-annotated major cell classes. **(B)** UMAP feature plot showing log-normalized MLPH expression in the same embedding. **(C)** Dot plot of canonical marker genes and MLPH across major cell classes. Dot color indicates scaled average expression, and dot size indicates the percentage of expressing cells. **(D)** MLPH expression across author-defined fine cell types stratified by tissue condition. Dot color indicates mean log-normalized MLPH expression, and dot size indicates the proportion of MLPH-positive cells. **(E)** Sample-level MLPH expression in epithelial compartments across tissue conditions. Each point represents one sample-level epithelial cell-type stratum; boxes show the median and interquartile range, with whiskers extending to 1.5 × the interquartile range. scRNA-seq, single-cell RNA sequencing; UMAP, Uniform Manifold Approximation and Projection.

To clarify the cellular context of the bulk transcriptomic signal, we examined MLPH expression in the prostate cancer scRNA-seq dataset GSE181294 (Figure 6). Using author-provided annotations, UMAP visualization and canonical marker expression identified the expected epithelial, immune, endothelial, fibroblast, and pericyte compartments (Figures 6A,C). MLPH expression was mainly localized to epithelial cells, with limited expression in immune and stromal populations (Figure 6B).

At fine cell-type resolution, tumor epithelial cells showed the highest mean MLPH expression and the largest proportion of MLPH-positive cells (Figure 6D). Non-malignant luminal and club epithelial cells showed lower but detectable expression, whereas most non-epithelial populations showed weak expression. Sample-level epithelial summaries confirmed that MLPH expression was highest in tumor epithelial cells (Figure 6E). These results indicate that the bulk-level MLPH signal is predominantly attributable to epithelial, particularly tumor epithelial, compartments.

### 3.9 Association of MLPH with clinical risk stratification and validated genomic prognostic signatures

**Figure 7:**
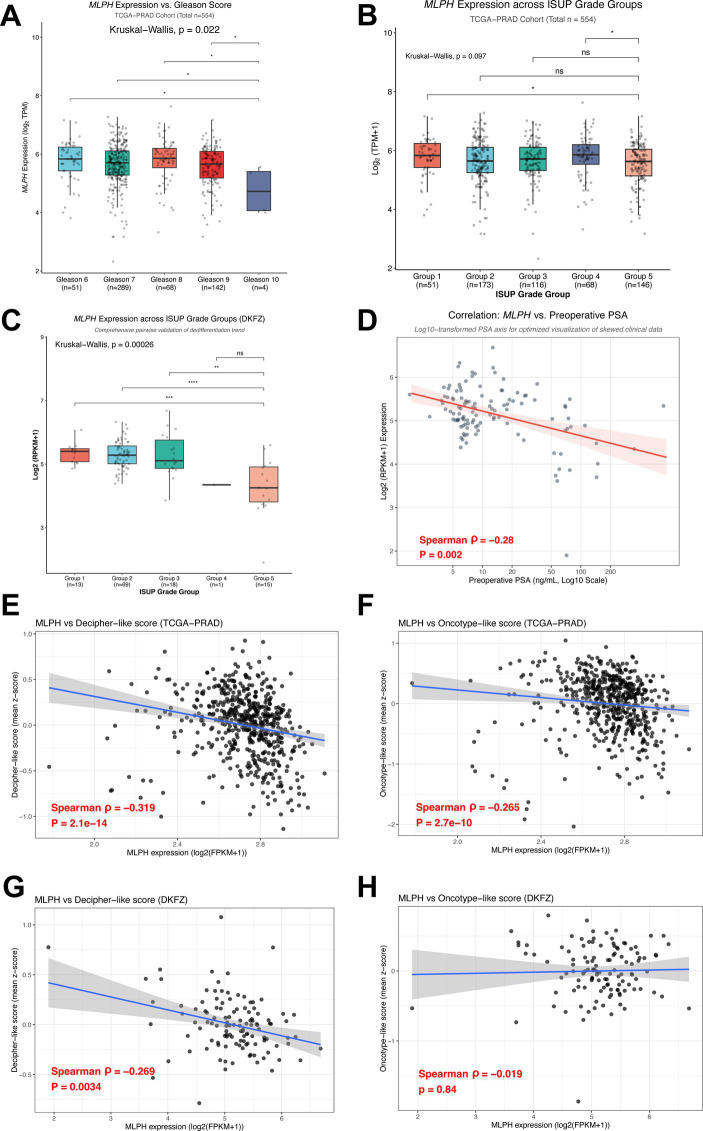
Association of MLPH expression with clinicopathological differentiation and genomic risk signatures. **(A)** Association between *MLPH* expression and Gleason scores in the TCGA-PRAD cohort (*n* = 554). A significant decline in *MLPH* expression was observed specifically in the Gleason 10 subgroup (*p* = 0.022, Kruskal-Wallis test). **(B)** *MLPH* expression across ISUP Grade Groups in the TCGA-PRAD cohort. Consistent with Gleason scoring, pairwise comparisons confirmed a significant downregulation in Grade Group 5 (GG5) relative to GG1 and GG4. **(C)** Independent validation in the DKFZ cohort (*n* = 116) showing significant variation across ISUP groups (*p* < 0.001), with a consistent reduction of *MLPH* in the highest-grade tumors (GG5). **(D)** Spearman correlation analysis in the DKFZ cohort reveals a significant negative association between *MLPH* expression and log10-transformed preoperative PSA levels (rho = −0.28, *p* = 0.002). **(E,F)** Evaluation of *MLPH* against genomic classifiers in the TCGA-PRAD cohort. *MLPH* expression correlates inversely with **(E)** the Decipher-like score (rho = −0.319, *p* = 2.1 × 10^−14^) and **(F)** the Oncotype DX-like score (rho = −0.265, *p* = 2.7 × 10^−10^), linking lower expression to higher genomic risk. **(G,H)** Validation in the DKFZ cohort. **(G)** The inverse correlation with the Decipher-like score was successfully replicated (rho = −0.269, *p* = 0.0034), while **(H)** the association with the Oncotype DX-like score did not reach statistical significance (rho = −0.019, *p* = 0.84). TCGA, The Cancer Genome Atlas; PRAD, prostate adenocarcinoma; ISUP, International Society of Urological Pathology.

Beyond verifying expression differences between tumor and normal tissues, we characterized the clinical relevance of *MLPH* by assessing its association with established prognostic frameworks in both the TCGA-PRAD discovery cohort (*n* = 554) and the independent DKFZ validation cohort (*n* = 116). In the TCGA-PRAD cohort, *MLPH* expression was significantly associated with Gleason scores (*p* = 0.022; Figure 7A). An initial inspection of the data revealed that while *MLPH* levels remained consistently high across Gleason 6–9, there was a notable reduction in the Gleason 10 subgroup. Targeted pairwise comparisons confirmed that *MLPH* expression in Gleason 10 tumors was significantly lower than in other high-grade subgroups. To align with modern pathological standards, we re-stratified the cohort into ISUP Grade Groups (1–5). Consistent with the Gleason score analysis, *MLPH* expression exhibited a specific decline in Grade Group 5 (GG5) compared to GG1 and GG4 (Figure 7B).

This “terminal drop” pattern was robustly validated in the independent DKFZ cohort, where *MLPH* showed significant global differences across ISUP groups (*p* < 0.001) and a prominent loss of expression in GG5 compared to early-stage groups (Figure 7C). Furthermore, correlation analysis with preoperative PSA—a key component of D’Amico and CAPRA risk scores—revealed a significant negative correlation (Spearman *R* = −0.28, *p* = 0.002; Figure 7D) in the DKFZ cohort, providing continuous evidence that *MLPH* levels are inversely linked to tumor burden in advanced stages. To further benchmark the prognostic relevance of *MLPH* against clinically established genomic risk assessment tools, we evaluated its association with the Decipher-like and Oncotype DX-like prognostic signatures. In the TCGA-PRAD cohort, *MLPH* expression displayed a robust inverse correlation with both the Decipher-like score (Spearman rho = −0.319, *p* = 2.1 × 10^−14^) and the Oncotype DX-like score (Spearman rho = −0.265, *p* = 2.7 × 10^−10^) (Figures 7E,F). We next validated these findings in an independent DKFZ prostate cancer cohort. In this cohort, the inverse association between *MLPH* expression and the Decipher-like score remained statistically significant, whereas the association with the Oncotype-like score showed a consistent negative direction but did not reach statistical significance (Figures 7G,H). As a supplementary outcome-level analysis, Kaplan–Meier and Cox regression analyses were performed in TCGA-PRAD and DKFZ cohorts with available follow-up data (Supplementary Figure S3). MLPH expression was not independently associated with TCGA-PRAD progression-free interval or DKFZ biochemical recurrence-free survival after clinicopathological adjustment, suggesting limited evidence for a robust independent survival effect in these cohorts.

### 3.10 Gene network analyses and pathway enrichment

**Figure 8:**
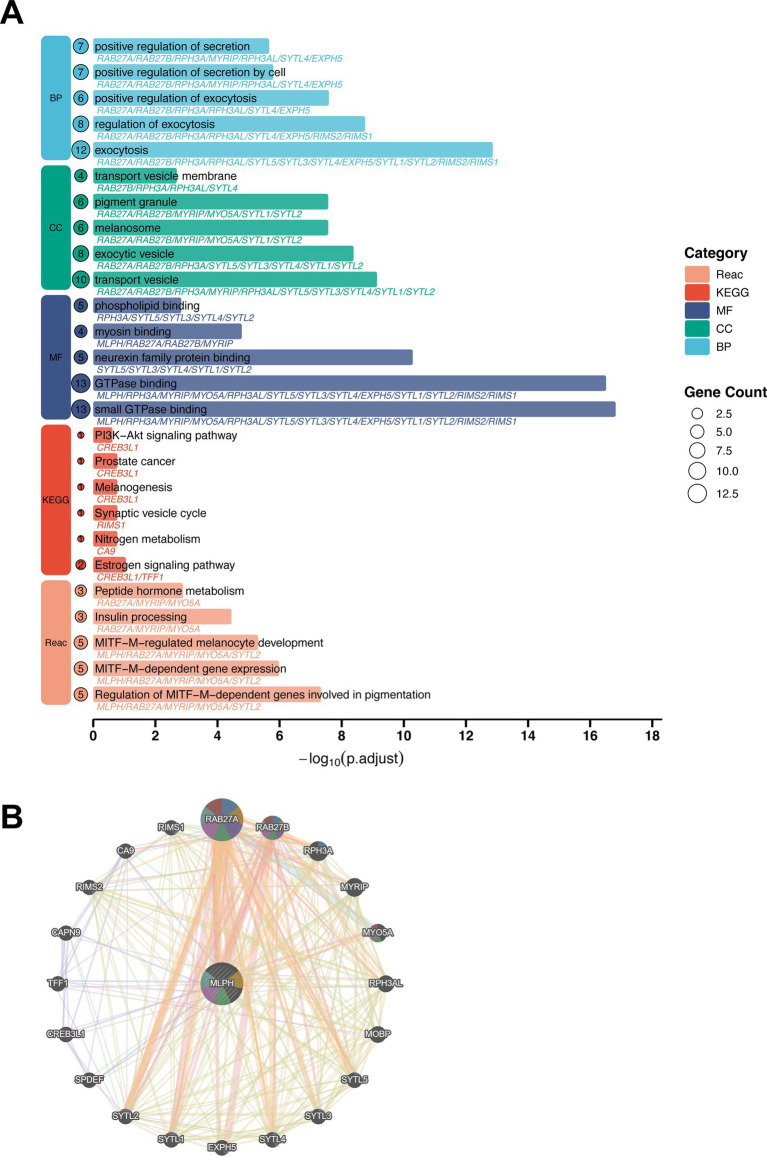
Functional enrichment and GeneMANIA gene network. **(A)** Functional enrichment analysis of 21 genes identified from the *MLPH* interaction network using GO, KEGG, and Reactome databases. **(B)** Gene interaction network generated using GeneMANIA. GO, Gene Ontology; KEGG, Kyoto Encyclopedia of Genes and Genomes.

The protein interaction network conducted by GeneMANIA platform (Figure 8B) revealed that *MLPH* physically interacts with several vesicle trafficking–related genes, including *RAB27A*, *RAB27B, MYO5A*, and multiple members of the *SYTL* family. In addition, colocalization relationships were identified between *MLPH* and cancer-relevant genes such as *TFF1* and *CAIX*, both previously implicated in prostate tumorigenesis (54–56). The most significantly enriched biological processes within this network included vesicle-mediated transport, pigment granule localization, and regulation of exocytosis (Supplementary Table S8), consistent with the transmembrane transport–related pathways highlighted by MAGMA-based enrichment analysis. Subsequent pathway enrichment analyses using GO, KEGG, and Reactome databases yielded consistent results (Figure 8A). GO analysis highlighted significant enrichment in terms such as exocytosis, transport vesicle, and small GTPase binding, the latter aligning with *MLPH’s* known function as an effector of *RAB27* GTPase. These pathways support the role of *MLPH* in coordinating vesicle docking and release events. In the KEGG database, pathways including the synaptic vesicle cycle, prostate cancer, and notably the estrogen signaling pathway were enriched, suggesting potential hormone-regulated vesicle transport mechanisms in prostate cancer progression. Reactome enrichment predominantly involved melanin biosynthesis and pigment granule transport–related pathways, consistent with the canonical role of *MLPH* in melanosome trafficking.

## 4 Discussion

In this study, we used an integrative genomic framework to prioritize candidate genes associated with PCa susceptibility. Cross-tissue and single-tissue TWAS, followed by conditional analysis, identified a set of transcriptome-wide signals with evidence of genetic association. Complementary gene-level analyses using MAGMA and fastBAT further refined these signals to 23 consensus candidate genes supported by multiple statistical approaches. Subsequent SMR and Bayesian colocalization analyses highlighted *MLPH* and *GGCX* as candidates with convergent evidence for genetically regulated expression and PCa risk. To further prioritize signals retained in the prostate cancer tumor context, we incorporated cis-eQTL evidence from TCGA-PRAD through PancanQTL. In this step, the MLPH lead variant rs7582964 showed a significant association with MLPH expression in PRAD tumor tissues, whereas *GGCX* did not show detectable tumor-context cis-eQTL support. *MLPH* was therefore selected for downstream characterization. Across transcriptomic and protein-level resources, MLPH showed higher expression in PCa tissues than in normal prostate tissues. Single-cell transcriptomic analysis further localized *MLPH* expression mainly to epithelial compartments, particularly tumor epithelial cells. Clinical annotation linked MLPH to tumor differentiation, preoperative PSA, and genomic risk signatures, whereas survival analyses did not support an independent recurrence-related effect after clinicopathological adjustment. Functional network and enrichment analyses further connected *MLPH* with vesicle-mediated transport, exocytosis, and hormone-related signaling. Together, these findings support *MLPH* as a genetically prioritized candidate gene with tumor-context regulatory and clinical annotation evidence in PCa.

Transcriptome-wide association studies has emerged as a powerful approach for identifying susceptibility genes in complex diseases. A previous PCa TWAS using the FUSION platform identified 217 candidate genes (57), but lacked further validation of genetic independence, putative causal links, and functional assessment at the transcriptomic and proteomic levels. Additionally, the biological context of these genes within regulatory networks and relevant pathways remained underexplored.

Based on integrative multi-omics analysis, *MLPH*, a gene encoding melanophilin, was identified as a potential risk gene for PCa. Prior studies utilizing chromosome conformation capture combined with immunoprecipitation have identified chromatin interactions linking *MLPH* to genomic loci associated with PCa risk (58). In a large-scale proteomic resource comprising over 18,000 transcripts and 12,000 proteins, *MLPH* received a Global Label Score of 3, reflecting consistent tissue-specific expression across multiple datasets (59). Notably, *MLPH* ranked among the top prostate-expressed genes or proteins in several datasets (60, 61). These results support a possible role for *MLPH* in prostate tissue biology and its relevance to prostate cancer pathogenesis.

*MLPH* encodes a critical component of the melanosome transport complex and has been linked to tumor progression in several malignancies, including breast, pancreatic and colorectal cancers (62–64). Our GeneMANIA analysis revealed direct physical interactions between *MLPH* and several key vesicle transport genes such as *RAB27A*, *MYO5A*, and members of the *SYTL* family. Functional enrichment further highlighted roles in vesicle-mediated transport, pigment granule localization, and exocytotic regulation. Given the established involvement of *RAB27A/B* in exosome secretion, including modulation of prostate-specific antigen and prostatic acid phosphatase via the *PI3K* pathway, it is plausible that *MLPH*—acting as a *RAB27* effector—participates in secretory mechanisms central to PCa pathogenesis (65, 66). Additionally, the co-localization of *MLPH* with known PCa-related genes such as *TFF1* and *CAIX* reinforces its potential functional relevance. *TFF1*, a stable secretory peptide, has been found elevated in PCa patient plasma and associated with adverse clinical features (55, 67). *CAIX*, a hypoxia-inducible enzyme, is overexpressed in acidic tumor environments, including PCa, and is implicated in extracellular acidification and metastasis (56).

Previous TWAS studies suggested that reduced *MLPH* expression in the prostate may be protective (57, 68), our study further confirmed this association in prostate using the FUSION single-tissue model (*Z* = −6.92, Bonferroni-adjusted *p* = 8.33 × 10^−8^). However, this signal did not pass COJO or SMR filtering, suggesting possible confounding from LD or non-independence of regulatory variants. Our study identified a significant positive association between *MLPH* expression and PCa risk in the testis tissue (*Z* = 4.31; *P*_SMR = 4.3 × 10^−6^; *p*_HEIDI > 0.01), this apparent discordance may reflect tissue-specific regulatory architectures or distal regulatory effects mediated by endocrine axes (69–71). Notably, testis-derived androgen production plays a central role in PCa biology, and *MLPH* expression in prostate tissues has been correlated with androgen receptor (AR) mRNA and protein levels (68, 72). Furthermore, *RAB27B*, a known upstream effector of *MLPH*, is positively correlated with AR expression, and *RAB27A* is reportedly regulated by androgen deprivation therapy (73). This correlation aligns with our clinical observations in the TCGA and DKFZ cohorts, where *MLPH* expression tracks with the luminal differentiation status. Most prostate cancers initiate as androgen-dependent acinar adenocarcinomas characterized by the expression of luminal markers like AR and PSA (74). In this context, the initial upregulation of *MLPH* likely reflects the expansion of the tumor-specific luminal population. However, our finding that *MLPH* expression drops in the most undifferentiated tumors (ISUP Grade 5) and negatively correlates with preoperative PSA suggests a lineage switch mechanism. As tumors progress toward an Aggressive Variant Prostate Cancer (AVPC) phenotype, they often undergo terminal dedifferentiation and lose luminal epithelial lineage factors, including AR and its downstream targets (75, 76). These findings suggest that *MLPH* may exert distinct roles depending on tissue context and disease stage: a locally suppressive effect in normal prostate epithelium, a driver of luminal expansion in typical adenocarcinoma, and a marker of dedifferentiation when lost in advanced disease. The opposite directions of association across tissues underscore the need for integrative approaches to resolve complex regulatory architectures in hormone-driven cancers.

Our study offers several notable advancements over previous TWAS efforts. We utilized UTMOST to incorporate multi-tissue expression models, applied stringent COJO criteria (joint significance and TOP.SNP.COR^2^ ≤ 0.6), and relied on updated GWAS (*n* = 726,828) and GTEx v8 datasets with 49% more RNA-seq samples than earlier versions (77). Furthermore, unlike previous studies that relied solely on statistical inference, we performed a comprehensive multi-cohort validation using TCGA, DKFZ, and GEO datasets. These data position MLPH as a candidate susceptibility gene related to tumor differentiation and genomic risk. The convergence of genetic, transcriptomic, and proteomic evidence strengthens the case for *MLPH* as a functionally relevant PCa-associated gene.

Several limitations should be acknowledged. First, the genetic discovery analyses were restricted to European-ancestry GWAS data and European-matched LD/eQTL reference resources. This design was chosen to reduce population stratification and LD mismatch in TWAS, SMR, and colocalization analyses, but it limits the direct generalizability of the findings. Because allele frequencies, LD structure, and regulatory effect sizes may differ across ancestries, the value of *MLPH* as a candidate biomarker should be tested in non-European and admixed populations using ancestry-matched eQTL resources and independent clinical cohorts. Second, although TCGA, DKFZ, GEO, HPA, and single-cell data provided convergent support for MLPH at the transcriptomic, protein, and cellular levels, these analyses remain based on public retrospective datasets. Differences in sampling, platform, clinical annotation, and tissue composition may influence the observed associations. Third, some GTEx tissues have limited sample sizes, which may reduce power for tissue-specific regulatory inference. Finally, our study prioritizes *MLPH* through statistical and bioinformatic evidence, but experimental studies are still needed to define its vesicle-transport functions and its role during prostate cancer progression.

## 5 Conclusion

Our integrative multi-omics framework identifies *MLPH* as a biologically plausible and statistically supported candidate gene for prostate cancer susceptibility. Its established roles in vesicle transport and interaction with secretory and AR-associated regulators suggest potential for *MLPH* as a therapeutic target worthy of further investigation. Furthermore, our multi-cohort clinical analysis demonstrates that *MLPH* expression is intimately associated with established prognostic indicators and genomic risk signatures, supporting its translational value as a candidate biomarker for future risk-stratification studies in prostate cancer.

## Data Availability

Publicly available datasets were analyzed in this study. This data can be found here: transcriptomic prediction models were obtained from the UTMOST repository (https://github.com/Joker-Jerome/UTMOST/), and eQTL reference panels were derived from GTEx V8 (https://ftp.ebi.ac.uk/pub/databases/spot/eQTL/imported/GTEx_V8/). GWAS summary statistics for prostate cancer were accessed via the GWAS Catalog (https://www.ebi.ac.uk/gwas/). TWAS analyses were conducted using the FUSION (http://gusevlab.org/projects/fusion/) and UTMOST pipelines. MAGMA gene-level analyses were performed using the MAGMA tool (https://cncr.nl/research/magma/), and SMR analyses were carried out using the SMR software (https://yanglab.westlake.edu.cn/software/smr/#SMR&HEIDIanalysis/). Immunohistochemical data were retrieved from the Human Protein Atlas (https://www.proteinatlas.org/), and gene interaction networks were constructed via GeneMANIA (https://genemania.org/). The specific analysis code and scripts used in this study are available at https://github.com/wryccmu/MLPH_ProstateCancer_Analysis.

## References

[1] Bray F Laversanne M Sung H Ferlay J Siegel RL Soerjomataram I . Global cancer statistics 2022: GLOBOCAN estimates of incidence and mortality worldwide for 36 cancers in 185 countries. CA Cancer J Clin. (2024) 74:229–63. doi: 10.3322/caac.21834, 38572751

[2] Siegel RL Kratzer TB Wagle NS Sung H Jemal A. Cancer statistics, 2026. CA Cancer J Clin. (2026) 76:e70043. doi: 10.3322/caac.70043, 41528114 PMC12798275

[3] Raychaudhuri R Lin DW Montgomery RB. Prostate Cancer: a review. JAMA. (2025) 333:1433–46. doi: 10.1001/jama.2025.0228, 40063046

[4] Lichtenstein P Holm NV Verkasalo PK Iliadou A Kaprio J Koskenvuo M . Environmental and heritable factors in the causation of cancer--analyses of cohorts of twins from Sweden, Denmark, and Finland. N Engl J Med. (2000) 343:78–85. doi: 10.1056/NEJM200007133430201, 10891514

[5] Baker SG Lichtenstein P Kaprio J Holm N. Genetic susceptibility to prostate, breast, and colorectal cancer among Nordic twins. Biometrics. (2005) 61:55–63. doi: 10.1111/j.0006-341X.2005.030924.x, 15737078

[6] Hjelmborg JB Scheike T Holst K Skytthe A Penney KL Graff RE . The heritability of prostate cancer in the Nordic twin study of Cancer. Cancer Epidemiol Biomarkers Prev. (2014) 23:2303–10. doi: 10.1158/1055-9965.EPI-13-0568, 24812039 PMC4221420

[7] Benafif S Kote-Jarai Z Eeles RA PRACTICAL Consortium. A review of prostate cancer genome-wide association studies (GWAS). Cancer Epidemiol Biomarkers Prev. (2018) 27:845–57. doi: 10.1158/1055-9965.EPI-16-1046, 29348298 PMC6051932

[8] Schumacher FR Al Olama AA Berndt SI Benlloch S Ahmed M Saunders EJ . Association analyses of more than 140,000 men identify 63 new prostate cancer susceptibility loci. Nat Genet. (2018) 50:928–36. doi: 10.1038/s41588-018-0142-8, 29892016 PMC6568012

[9] Maurano MT Humbert R Rynes E Thurman RE Haugen E Wang H . Systematic localization of common disease-associated variation in regulatory DNA. Science. (2012) 337:1190–5. doi: 10.1126/science.1222794, 22955828 PMC3771521

[10] Tam V Patel N Turcotte M Bossé Y Paré G Meyre D. Benefits and limitations of genome-wide association studies. Nat Rev Genet. (2019) 20:467–84. doi: 10.1038/s41576-019-0127-1, 31068683

[11] Gamazon ER Wheeler HE Shah KP Mozaffari SV Aquino-Michaels K Carroll RJ . A gene-based association method for mapping traits using reference transcriptome data. Nat Genet. (2015) 47:1091–8. doi: 10.1038/ng.3367, 26258848 PMC4552594

[12] Hu Y Li M Lu Q Weng H Wang J Zekavat SM . A statistical framework for cross-tissue transcriptome-wide association analysis. Nat Genet. (2019) 51:568–76. doi: 10.1038/s41588-019-0345-7, 30804563 PMC6788740

[13] Gui J Yang X Tan C Wang L Meng L Han Z . A cross-tissue transcriptome-wide association study reveals novel susceptibility genes for migraine. J Headache Pain. (2024) 25:94. doi: 10.1186/s10194-024-01802-6, 38840241 PMC11151630

[14] Yang H Huang H Pu K. A cross-tissue transcriptome-wide association study identified susceptibility genes for age-related macular degeneration. Sci Rep. (2025) 15:4788. doi: 10.1038/s41598-025-89246-z, 39922885 PMC11807202

[15] Zhu T Ma Y Yang P Cao Z Gao J Du J . A cross-tissue transcriptome-wide association study reveals novel susceptibility genes for erectile dysfunction. Andrology. (2025) 14:159–68. doi: 10.1111/andr.70034, 40145662

[16] Zhou X Ye X Yao J Lin X Weng Y Huang Y . Identification and validation of transcriptome-wide association study-derived genes as potential druggable targets for osteoarthritis. Bone Joint Res. (2025) 14:224–35. doi: 10.1302/2046-3758.143.BJR-2024-0251.R1, 40079200 PMC11904851

[17] Hu T Parrish RL Dai Q Buchman AS Tasaki S Bennett DA . Omnibus proteome-wide association study identifies 43 risk genes for Alzheimer disease dementia. Am J Hum Genet. (2024) 111:1848–63. doi: 10.1016/j.ajhg.2024.07.001, 39079537 PMC11393696

[18] GTEx Consortium Laboratory, Data Analysis & Coordinating Center (LDACC)-Analysis Working Group Statistical Methods groups-Analysis Working Group Enhancing GTEx (eGTEx) groups NIH Common Fund NIH/NCI . Genetic effects on gene expression across human tissues. Nature. (2017) 550:204–13. doi: 10.1038/nature24277 29022597 PMC5776756

[19] Zhu Z Zhang F Hu H Bakshi A Robinson MR Powell JE . Integration of summary data from GWAS and eQTL studies predicts complex trait gene targets. Nat Genet. (2016) 48:481–7. doi: 10.1038/ng.3538, 27019110

[20] Giambartolomei C Vukcevic D Schadt EE Franke L Hingorani AD Wallace C . Bayesian test for colocalisation between pairs of genetic association studies using summary statistics. PLoS Genet. (2014) 10:e1004383. doi: 10.1371/journal.pgen.1004383, 24830394 PMC4022491

[21] Wang A Shen J Rodriguez AA Saunders EJ Chen F Janivara R . Characterizing prostate cancer risk through multi-ancestry genome-wide discovery of 187 novel risk variants. Nat Genet. (2023) 55:2065–74. doi: 10.1038/s41588-023-01534-4, 37945903 PMC10841479

[22] Sun R Hui S Bader GD Lin X Kraft P. Powerful gene set analysis in GWAS with the generalized Berk-Jones statistic. PLoS Genet. (2019) 15:e1007530. doi: 10.1371/journal.pgen.1007530, 30875371 PMC6436759

[23] Gusev A Ko A Shi H Bhatia G Chung W Penninx BWJH . Integrative approaches for large-scale transcriptome-wide association studies. Nat Genet. (2016) 48:245–52. doi: 10.1038/ng.3506, 26854917 PMC4767558

[24] Pennisi E. Genomics. 1000 genomes project gives new map of genetic diversity. Science. (2010) 330:574–5. doi: 10.1126/science.330.6004.574 21030618

[25] Li SJ Shi JJ Mao CY Zhang C Xu YF Fan Y . Identifying causal genes for migraine by integrating the proteome and transcriptome. J Headache Pain. (2023) 24:111. doi: 10.1186/s10194-023-01649-3, 37592229 PMC10433568

[26] Cloney R. Complex traits: integrating gene variation and expression to understand complex traits. Nat Rev Genet. (2016) 17:194. doi: 10.1038/nrg.2016.18 26900024

[27] Liao C Laporte AD Spiegelman D Akçimen F Joober R Dion PA . Transcriptome-wide association study of attention deficit hyperactivity disorder identifies associated genes and phenotypes. Nat Commun. (2019) 10:4450. doi: 10.1038/s41467-019-12450-9, 31575856 PMC6773763

[28] de Leeuw CA Mooij JM Heskes T Posthuma D. MAGMA: generalized gene-set analysis of GWAS data. PLoS Comput Biol. (2015) 11:e1004219. doi: 10.1371/journal.pcbi.1004219, 25885710 PMC4401657

[29] de Leeuw CA Stringer S Dekkers IA Heskes T Posthuma D. Conditional and interaction gene-set analysis reveals novel functional pathways for blood pressure. Nat Commun. (2018) 9:3768. doi: 10.1038/s41467-018-06022-6, 30218068 PMC6138636

[30] de Leeuw CA Neale BM Heskes T Posthuma D. The statistical properties of gene-set analysis. Nat Rev Genet. (2016) 17:353–64. doi: 10.1038/nrg.2016.29, 27070863

[31] Chen G Jin Y Chu C Zheng Y Yang C Chen Y . A cross-tissue transcriptome-wide association study reveals GRK4 as a novel susceptibility gene for COPD. Sci Rep. (2024) 14:28438. doi: 10.1038/s41598-024-80122-w, 39558015 PMC11574126

[32] Yang J Lee SH Goddard ME Visscher PM. GCTA: a tool for genome-wide complex trait analysis. Am J Hum Genet. (2011) 88:76–82. doi: 10.1016/j.ajhg.2010.11.011, 21167468 PMC3014363

[33] Bakshi A Zhu Z Vinkhuyzen AAE Hill WD McRae AF Visscher PM . Fast set-based association analysis using summary data from GWAS identifies novel gene loci for human complex traits. Sci Rep. (2016) 6:32894. doi: 10.1038/srep32894, 27604177 PMC5015118

[34] Guo P Gong W Li Y Liu L Yan R Wang Y . Pinpointing novel risk loci for Lewy body dementia and the shared genetic etiology with Alzheimer’s disease and Parkinson’s disease: a large-scale multi-trait association analysis. BMC Med. (2022) 20:214. doi: 10.1186/s12916-022-02404-2, 35729600 PMC9214990

[35] Qi T Wu Y Zeng J Zhang F Xue A Jiang L . Identifying gene targets for brain-related traits using transcriptomic and methylomic data from blood. Nat Commun. (2018) 9:2282. doi: 10.1038/s41467-018-04558-1, 29891976 PMC5995828

[36] Yao P Mazidi M Pozarickij A Iona A Wright N Lin K . Proteome-wide genetic study in east Asians and Europeans identified multiple therapeutic targets for ischemic stroke. Stroke. (2025) 56:2147–58. doi: 10.1161/STROKEAHA.125.050982, 40304040

[37] Chen C Liu Y Luo M Yang J Chen Y Wang R . PancanQTLv2.0: a comprehensive resource for expression quantitative trait loci across human cancers. Nucleic Acids Res. (2024) 52:D1400–6. doi: 10.1093/nar/gkad916, 37870463 PMC10767806

[38] Goldman MJ Craft B Hastie M Repečka K McDade F Kamath A . Visualizing and interpreting cancer genomics data via the Xena platform. Nat Biotechnol. (2020) 38:675–8. doi: 10.1038/s41587-020-0546-8, 32444850 PMC7386072

[39] Love MI Huber W Anders S. Moderated estimation of fold change and dispersion for RNA-seq data with DESeq2. Genome Biol. (2014) 15:550. doi: 10.1186/s13059-014-0550-8, 25516281 PMC4302049

[40] Ridlon JM Devendran S Alves JM Doden H Wolf PG Pereira GV . The “in vivo lifestyle” of bile acid 7α-dehydroxylating bacteria: comparative genomics, metatranscriptomic, and bile acid metabolomics analysis of a defined microbial community in gnotobiotic mice. Gut Microbes. (2020) 11:381–404. doi: 10.1080/19490976.2019.1618173, 31177942 PMC7524365

[41] Uhlén M Fagerberg L Hallström BM Lindskog C Oksvold P Mardinoglu A . Proteomics. Tissue-based map of the human proteome. Science. (2015) 347:1260419. doi: 10.1126/science.1260419, 25613900

[42] Jain Y Godwin LL Joshi S Mandarapu S le T Lindskog C . Segmenting functional tissue units across human organs using community-driven development of generalizable machine learning algorithms. Nat Commun. (2023) 14:4656. doi: 10.1038/s41467-023-40291-0, 37537179 PMC10400613

[43] Schindelin J Arganda-Carreras I Frise E Kaynig V Longair M Pietzsch T . Fiji: an open-source platform for biological-image analysis. Nat Methods. (2012) 9:676–82. doi: 10.1038/nmeth.2019, 22743772 PMC3855844

[44] Hirz T Mei S Sarkar H Kfoury Y Wu S Verhoeven BM . Dissecting the immune suppressive human prostate tumor microenvironment via integrated single-cell and spatial transcriptomic analyses. Nat Commun. (2023) 14:663. doi: 10.1038/s41467-023-36325-2, 36750562 PMC9905093

[45] Gerhauser C Favero F Risch T Simon R Feuerbach L Assenov Y . Molecular evolution of early-onset prostate Cancer identifies molecular risk markers and clinical trajectories. Cancer Cell. (2018) 34:996–1011.e8. doi: 10.1016/j.ccell.2018.10.016, 30537516 PMC7444093

[46] Epstein JI Egevad L Amin MB Delahunt B Srigley JR Humphrey PA . The 2014 International Society of Urological Pathology (ISUP) consensus conference on Gleason grading of prostatic carcinoma: definition of grading patterns and proposal for a new grading system. Am J Surg Pathol. (2016) 40:244–52. doi: 10.1097/PAS.0000000000000530, 26492179

[47] Lin DW Zheng Y McKenney JK Brown MD Lu R Crager M . 17-gene genomic prostate score test results in the canary prostate active surveillance study (PASS) cohort. J Clin Oncol. (2020) 38:1549–57. doi: 10.1200/JCO.19.02267, 32130059 PMC7213589

[48] Spratt DE Yousefi K Deheshi S Ross AE den RB Schaeffer EM . Individual patient-level Meta-analysis of the performance of the decipher genomic classifier in high-risk men after prostatectomy to predict development of metastatic disease. J Clin Oncol. (2017) 35:1991–8. doi: 10.1200/JCO.2016.70.2811, 28358655 PMC6530581

[49] Mostafavi S Ray D Warde-Farley D Grouios C Morris Q. GeneMANIA: a real-time multiple association network integration algorithm for predicting gene function. Genome Biol. (2008) 9:S4. doi: 10.1186/gb-2008-9-s1-s4, 18613948 PMC2447538

[50] Wu T Hu E Xu S Chen M Guo P Dai Z . clusterProfiler 4.0: a universal enrichment tool for interpreting omics data. Innovation (Camb). (2021) 2:100141. doi: 10.1016/j.xinn.2021.100141, 34557778 PMC8454663

[51] Gene Ontology Consortium. Gene ontology consortium: going forward. Nucleic Acids Res. (2015) 43:D1049–56. doi: 10.1093/nar/gku1179 25428369 PMC4383973

[52] Kanehisa M Furumichi M Sato Y Kawashima M Ishiguro-Watanabe M. KEGG for taxonomy-based analysis of pathways and genomes. Nucleic Acids Res. (2023) 51:D587–92. doi: 10.1093/nar/gkac963, 36300620 PMC9825424

[53] Milacic M Beavers D Conley P Gong C Gillespie M Griss J . The Reactome pathway knowledgebase 2024. Nucleic Acids Res. (2024) 52:D672–8. doi: 10.1093/nar/gkad1025, 37941124 PMC10767911

[54] Martín-Martín N Zabala-Letona A Fernández-Ruiz S Arreal L Camacho L Castillo-Martin M . PPARδ elicits ligand-independent repression of trefoil factor family to limit prostate Cancer growth. Cancer Res. (2018) 78:399–409. doi: 10.1158/0008-5472.CAN-17-0908, 29187400

[55] Vestergaard EM Borre M Poulsen SS Nexø E Tørring N. Plasma levels of trefoil factors are increased in patients with advanced prostate cancer. Clin Cancer Res. (2006) 12:807–12. doi: 10.1158/1078-0432.CCR-05-1545, 16467092

[56] Logozzi M Capasso C Di Raimo R Del Prete S Mizzoni D Falchi M . Prostate cancer cells and exosomes in acidic condition show increased carbonic anhydrase IX expression and activity. J Enzyme Inhib Med Chem. (2019) 34:272–8. doi: 10.1080/14756366.2018.1538980, 30734594 PMC6327996

[57] Mancuso N Gayther S Gusev A Zheng W Penney KL Kote-Jarai Z . Large-scale transcriptome-wide association study identifies new prostate cancer risk regions. Nat Commun. (2018) 9:4079. doi: 10.1038/s41467-018-06302-1, 30287866 PMC6172280

[58] Giambartolomei C Seo JH Schwarz T Freund MK Johnson RD Spisak S . H3K27ac HiChIP in prostate cell lines identifies risk genes for prostate cancer susceptibility. Am J Hum Genet. (2021) 108:2284–300. doi: 10.1016/j.ajhg.2021.11.007, 34822763 PMC8715276

[59] Malmström E Malmström L Hauri S Mohanty T Scott A Karlsson C . Human proteome distribution atlas for tissue-specific plasma proteome dynamics. Cell. (2025) 188:2810–2822.e16. doi: 10.1016/j.cell.2025.03.013, 40203824

[60] Jiang L Wang M Lin S Jian R Li X Chan J . A quantitative proteome map of the human body. Cell. (2020) 183:269–283.e19. doi: 10.1016/j.cell.2020.08.036, 32916130 PMC7575058

[61] Moreno P Fexova S George N Manning JR Miao Z Mohammed S . Expression atlas update: gene and protein expression in multiple species. Nucleic Acids Res. (2022) 50:D129–40. doi: 10.1093/nar/gkab1030, 34850121 PMC8728300

[62] Yao X Yuen T Qingchuan C Jianjun Z Yefu L Shulan S. Melanophilin inhibit the growth and lymph node metastasis of triple negative breast cancer via the NONO-SPHK1-S1P axis. J Transl Med. (2025) 23:284. doi: 10.1186/s12967-025-06240-9, 40050909 PMC11887221

[63] Chao YY Lin RC Su PJ Wang CA Tu TY Hou YC . Melanophilin-induced primary cilia promote pancreatic cancer metastasis. Cell Death Dis. (2025) 16:22. doi: 10.1038/s41419-025-07344-2, 39820281 PMC11739566

[64] Li WS Chen CI Chen HP Liu KW Tsai CJ Yang CC. Overexpression of MLPH in rectal Cancer patients correlates with a poorer response to preoperative Chemoradiotherapy and reduced patient survival. Diagnostics (Basel). (2021) 11:2132. doi: 10.3390/diagnostics11112132, 34829479 PMC8621396

[65] Zheng Y Campbell EC Lucocq J Riches A Powis SJ. Monitoring the Rab27 associated exosome pathway using nanoparticle tracking analysis. Exp Cell Res. (2013) 319:1706–13. doi: 10.1016/j.yexcr.2012.10.006, 23092844

[66] Johnson JL Ellis BA Noack D Seabra MC Catz SD. The Rab27a-binding protein, JFC1, regulates androgen-dependent secretion of prostate-specific antigen and prostatic-specific acid phosphatase. Biochem J. (2005) 391:699–710. doi: 10.1042/BJ20050380, 16004602 PMC1276972

[67] Hoffmann W Jagla W Wiede A. Molecular medicine of TFF-peptides: from gut to brain. Histol Histopathol. (2001) 16:319–34. doi: 10.14670/HH-16.319, 11193208

[68] Bu H Narisu N Schlick B Rainer J Manke T Schäfer G . Putative prostate Cancer risk SNP in an androgen receptor-binding site of the Melanophilin gene illustrates enrichment of risk SNPs in androgen receptor target sites. Hum Mutat. (2016) 37:52–64. doi: 10.1002/humu.22909, 26411452 PMC4715509

[69] Mitsiades N. A road map to comprehensive androgen receptor axis targeting for castration-resistant prostate cancer. Cancer Res. (2013) 73:4599–605. doi: 10.1158/0008-5472.CAN-12-4414, 23887973

[70] Cao Q Song Z Ruan H Wang C Yang X Bao L . Targeting the KIF4A/AR Axis to reverse endocrine therapy resistance in castration-resistant prostate Cancer. Clin Cancer Res. (2020) 26:1516–28. doi: 10.1158/1078-0432.CCR-19-0396, 31796514

[71] Xu F Shi J Qin X Zheng Z Chen M Lin Z . Hormone-glutamine metabolism: a critical regulatory Axis in endocrine-related cancers. Int J Mol Sci. (2022) 23:10086. doi: 10.3390/ijms231710086, 36077501 PMC9456462

[72] Takeda DY Spisák S Seo JH Bell C O’Connor E Korthauer K . A somatically acquired enhancer of the androgen receptor is a noncoding driver in advanced prostate Cancer. Cell. (2018) 174:422–432.e13. doi: 10.1016/j.cell.2018.05.037, 29909987 PMC6046260

[73] Shaw GL Whitaker H Corcoran M Dunning MJ Luxton H Kay J . The early effects of rapid androgen deprivation on human prostate Cancer. Eur Urol. (2016) 70:214–8. doi: 10.1016/j.eururo.2015.10.042, 26572708 PMC4926724

[74] Delgado C Mendez-Callejas G Celis C. Caryophyllene oxide, the active compound isolated from leaves of *Hymenaea courbaril* L. (Fabaceae) with antiproliferative and apoptotic effects on PC-3 androgen-independent prostate cancer cell line. Molecules. (2021) 26:6142. doi: 10.3390/molecules26206142 34684723 PMC8538860

[75] Davies A Zoubeidi A Beltran H Selth LA. The transcriptional and epigenetic landscape of Cancer cell lineage plasticity. Cancer Discov. (2023) 13:1771–88. doi: 10.1158/2159-8290.CD-23-0225, 37470668 PMC10527883

[76] Kouroukli O Bravou V Giannitsas K Tzelepi V. Tissue-based diagnostic biomarkers of aggressive variant prostate Cancer: a narrative review. Cancers (Basel). (2024) 16:805. doi: 10.3390/cancers16040805, 38398199 PMC10887410

[77] GTEx Consortium. The GTEx consortium atlas of genetic regulatory effects across human tissues. Science. (2020) 369:1318–30. doi: 10.1126/science.aaz1776, 32913098 PMC7737656

