# Supplementary material for: Integrative genomic profiling identifies MLPH as a candidate gene in prostate cancer
Source: Front Med (Lausanne). 2026 Jul 10;13:1878505. doi: 10.3389/fmed.2026.1878505 (PMC13395884; doi:10.3389/fmed.2026.1878505)

**Supplementary Figure 1. Tissue and pathway enrichment results based on MAGMA gene-level associations**


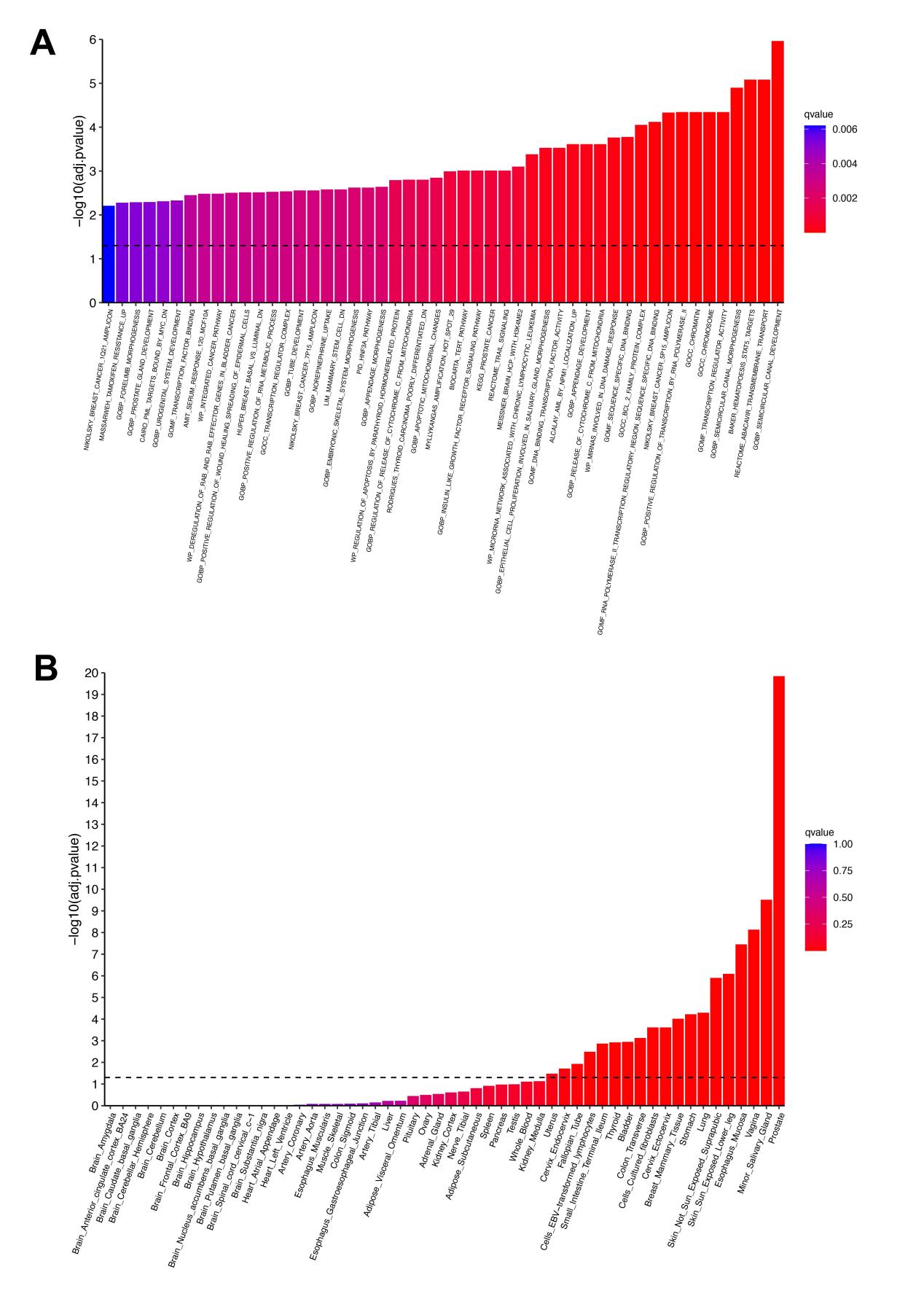


Abbreviations: MAGMA, Multi-marker Analysis of Genomic Annotation.

**Supplementary Figure 2. Colocalization plots for SMR-significant genes without evidence of colocalization**


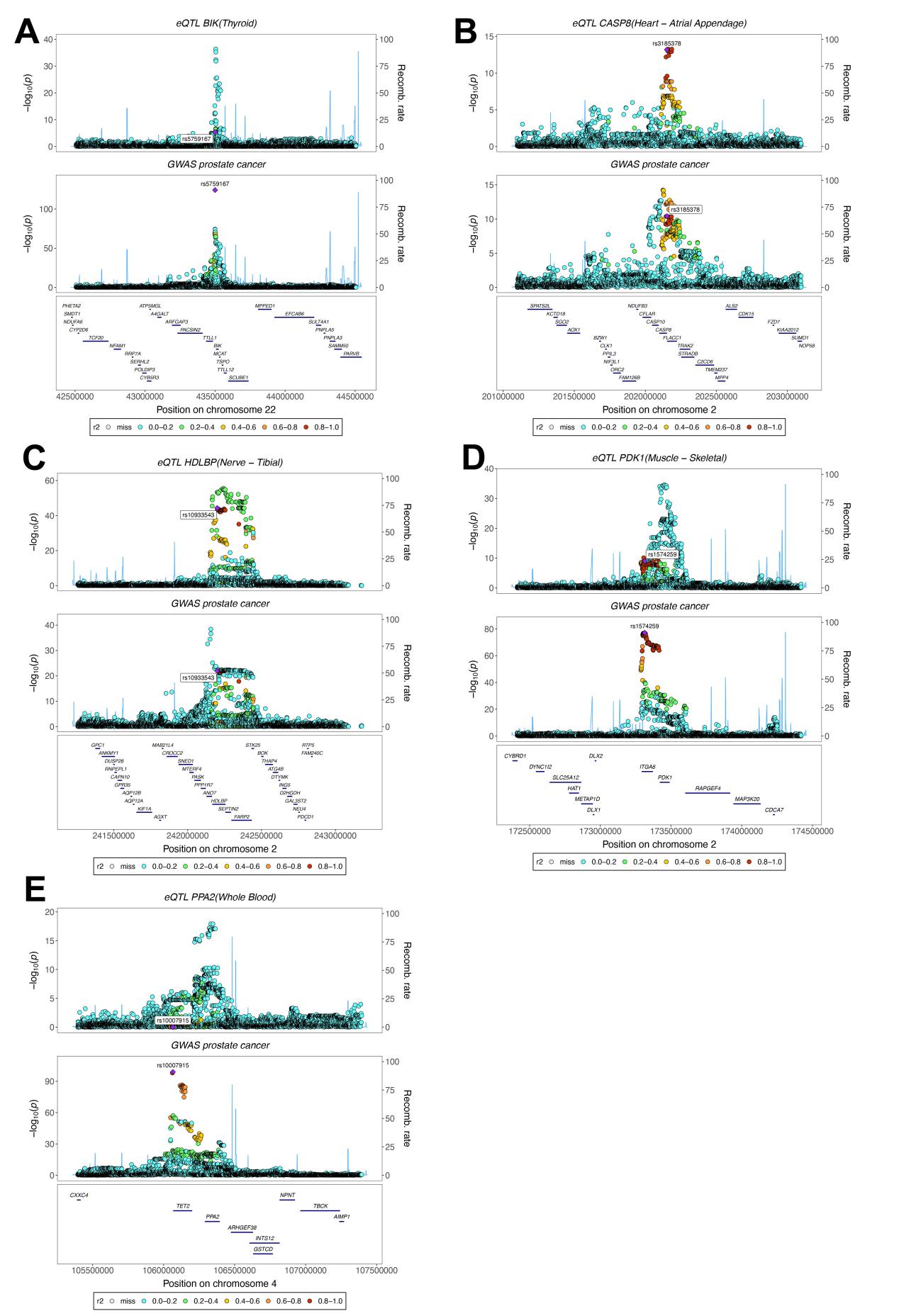


Abbreviations: GWAS, genome-wide association studies; eQTL, expression quantitative trait loci.

**Supplementary Figure 3. Prognostic evaluation of MLPH expression in TCGA-PRAD and DKFZ prostate cancer cohorts**


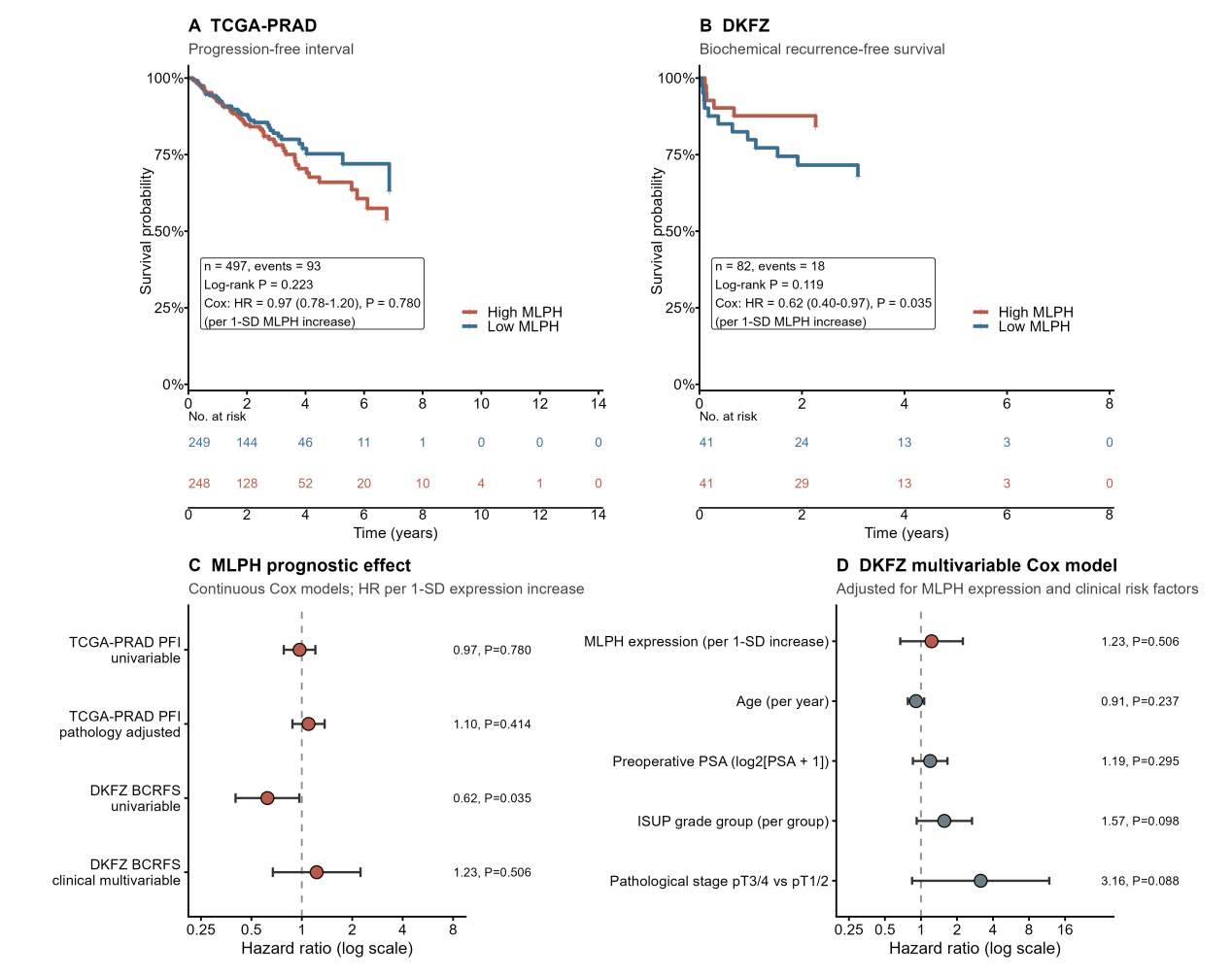

Supplement: Supplementary file 1 [file Table_1.DOCX]
